# Growth Arrest Specific 1 (*Gas1*) Gene Overexpression in Liver Reduces the *In Vivo* Progression of Murine Hepatocellular Carcinoma and Partially Restores Gene Expression Levels

**DOI:** 10.1371/journal.pone.0132477

**Published:** 2015-07-10

**Authors:** Natalia Sacilotto, Josefa Castillo, Ángela L. Riffo-Campos, Juana M. Flores, Olivia Hibbitt, Richard Wade-Martins, Carlos López, M. Isabel Rodrigo, Luis Franco, Gerardo López-Rodas

**Affiliations:** 1 Department of Biochemistry and Molecular Biology, University of Valencia, Burjassot, Valencia, Spain; 2 Institute of Health Research INCLIVA, Valencia, Spain; 3 Department of Medicine and Animal Surgery, University Complutense, Madrid, Spain; 4 Department of Physiology, Anatomy and Genetics, Oxford University, Oxford, United Kingdom; 5 Department of Cell Biology, University of Valencia, Burjassot, Valencia, Spain; University of Navarra School of Medicine and Center for Applied Medical Research (CIMA), SPAIN

## Abstract

The prognosis of hepatocellular carcinoma patients is usually poor, the size of tumors being a limiting factor for surgical treatments. Present results suggest that the overexpression of *Gas1* (growth arrest specific 1) gene reduces the size, proliferating activity and malignancy of liver tumors. Mice developing diethylnitrosamine-induced hepatocellular carcinoma were subjected to hydrodynamic gene delivery to overexpress *Gas1* in liver. This treatment significantly (p < 0.05) reduced the number of large tumors, while the difference in the total number of lesions was not significant. Moreover, the number of carcinoma foci in the liver and the number of lung metastases were reduced. These results are related with the finding that overexpression of *Gas1* in Hepa 1-6 cells arrests cell cycle before S phase, with a significant (p < 0.01) and concomitant reduction in the expression of cyclin E2 gene. In addition, a triangular analysis of microarray data shows that *Gas1* overexpression restores the transcription levels of 150 genes whose expression was affected in the diethylnitrosamine-induced tumors, thirteen of which are involved in the hedgehog signaling pathway. Since the *in vivo Gas1* gene delivery to livers of mice carrying hepatocellular carcinoma reduces the size and proliferating activity of tumors, partially restoring the transcriptional profile of the liver, the present study opens promising insights towards a therapeutic approach for hepatocellular carcinoma.

## Introduction

Hepatocellular carcinoma (HCC) is the sixth most common malignancy worldwide and the third major cause of deaths attributable to cancer [1]. The prognosis of HCC patients is usually poor, the size of tumors being a limit to surgical resection and liver transplantation [2] and only patients with small tumors are candidates for resection or local ablation [3]. Moreover, the risk of vascular dissemination increases with tumor size [1]. Therefore, attempts to reduce the size and proliferating activity of liver tumors constitute an attractive strategy prior to resection.

The growth arrest specific (*Gas1*) gene, which codes for a glycosylphosphatidylinositol-anchored protein [4], was first described in a search for genes preferentially expressed in growth-arrested cells [5], and its overexpression results in the arrest of the cell cycle before S phase [6]. The localization of human *GAS1* to chromosomal bands 9q21.3-q22, often deleted in myeloid malignancies, suggested that this gene could be involved in the suppression of tumor cell proliferation [7]

Del Sal et al. [8] first showed that overexpression of human *GAS1* arrested the proliferation of cell lines from lung and bladder carcinoma and similar results were obtained using a variety of other cell lines [9–12]. Correlations between proliferating activity and *GAS1* expression were found in thyroid [13] and gastric [12] tumors, as well as in mesangial cells [14]. Also, the expression of *GAS1* has been included in a test to discriminate between prostate cancer and benign tumors [15], its level being a prediction marker for metastasis or recurrence in stages II and III of colorectal cancer [16].

The potential role of *GAS1* as a tumor suppressor was studied by analyzing tumor progression after the implantation of *Gas1*-overexpressing malignant cells in mice. This study reported that the number and growth rate of the induced tumors in animals overexpressing *Gas1* were significantly lower than in control animals [10]. Zamorano et al. [17] inoculated a group of athymic mice with C6 glioma cells and another group with retrovirus-producing C6 glioma cells carrying either the human *GAS1* gene under the control of a glial-specific promoter or an unrelated gene. The volume of tumors was significantly smaller in mice expressing *Gas1* than in those of the other groups. Similar results, using a different experimental strategy, were afterwards reported by the same laboratory [18]. Recent results from the same group show that tGAS1, a truncated, soluble form of the protein, inhibits proliferation and angiogenesis in a triple negative breast cancer model [19].

The mechanisms by which *GAS1* gene exerts its antitumor activity remain largely unknown. It has been suggested that GAS1 mediates hedgehog signaling [20–24], one of the pathways involved in cancer development and metastasis as well as in the maintenance of the cancer stem cell phenotype (for a review, see [25]). It has also been hypothesized that GAS1 modifies the RET signaling pathway [26], also involved in cell growth and proliferation.

In summary, the current available data suggest that overexpression of *GAS1* might represent a potential antitumor therapeutic approach, although it has never been assessed on primary tumors *in vivo*. In the present paper, we show that the *in vivo* overexpression of *Gas1* in livers reduces the size and proliferating activity of HCC primary tumors as well as the number of lung metastases in mice. Additionally, microarray analysis showed that transfection with *Gas1* causes the expression of many liver genes to revert to their non-tumor level. In this regard, the potential therapeutic use of *Gas1* may be considered.

## Materials and Methods

### Materials

The following antibodies were used: rat monoclonal anti-hemagglutinin (HA) (Roche, Basel, Switzerland #11.867.423.001); mouse monoclonal anti-bromodeoxyuridine (BrdU) (Dako, Glostrup, Denmark, #M0744); goat anti-mouse GAS1 (R&D Systems, Minneapolis, MN, #AF2644); mouse anti-goat IgG Alexa fluor 555 (Invitrogen, Carlsbad, CA, #21425); goat anti-mouse IgG Alexa Fluor 488 (Invitrogen, #11001); goat anti-mouse IgG Alexa Fluor 594 (Invitrogen #11005); goat anti-rat IgG Alexa Fluor 488 (Invitrogen #11006). The plasmids used in this work were: pCAG-H2BGFP, expression vector coding for the fusion protein H2B-green fluorescent protein (GFP) under the control of CAG promoter (a gift from George Trichas, University of Oxford); plux.promoter^-^, containing the luciferase gene, without promoter; pCMV.lux, expression vector containing the luciferase gene under the control of cytomegalovirus (CMV) promoter (this work); pCAG.lux, expression plasmid containing the luciferase gene under the control of CAG promoter (this work); pcDNA3/CMV-HAGas1, expression vector designed to express an HA-N-tagged GAS1 fusion protein under the CMV promoter (this work); pcDNA3/CAG-HAGas1, expression vector designed to express an HA-N-tagged GAS1 fusion protein under the CAG promoter (this work); pIRES/HAGas1, bicistronic expression vector, coding for the fusion protein HA-GAS1 and the GFP, under the CMV promoter (this work); pcDNA3.1/V5-His-TOPO/lacZ, expression vector coding for the β-galactosidase, with a V5 epitope on its N-terminus, and a His tag on the C-terminus (Invitrogen).

### Cell culture and transfection methods

The mouse hepatoma cell line Hepa 1–6 (*ATCC CRL-1830*) was used for the *in vitro* experiments. Cells were grown at 37°C in a humid atmosphere containing 5% CO_2_ in high glucose Dulbecco modified Eagle’s medium supplemented with 10% heat-inactivated fetal bovine serum (Gibco, Invitrogen), 100 U/ml penicillin, 100 μg/ml streptomycin and 2.5 μg/ml fungizone (Gibco). Cells were transfected with Lipofectamine 2000 reagent (Invitrogen), following the manufacturer’s instructions.

### Animals

Eight to ten week-old CD1 male mice were obtained from the animal facilities of the University of Valencia, and fed *ad libitum* with standard diet. All procedures involving live animals were conducted under isofluorane inhalational anesthesia in a properly equipped surgical theatre, in accordance with the European regulations (Council Directive 86/609/EEC) and were authorized by the Ethics Committee for Animal Experimentation of the University of Valencia (approval of procedure for project BFU2007-63120, date 12/03/2007). Hydrodynamic gene delivery (HGD) was performed as described by Liu et al. [27], using 50 μg of plasmid DNA.

### Cell sorting and flow cytometry analysis

For cell sorting, suspensions of 3×10^6^ cells/ml in growth medium were used. Non transfected cells were used to define the region of auto-fluorescence, and 10^6^ transfected cells were recovered after sorting (MoFlo High Performance Cell Sorter, Dako Cytomation, Glostrup, Denmark). These cells were analyzed after propidium iodide staining in an Epics XL-MCL cytometer (Beckman Coulter, Brea, CA, USA). Transfection efficiency of cells with IRES (internal ribosome entry site) bicistronic plasmids was determined by measuring the green fluorescence emission.

### HCC development

Mice were intraperitoneally injected 15 days after birth with 25 μg DEN (diethyl nitrosamine) per g body weight, and kept under standard conditions until sacrifice. Tumors in the two major lobes of the liver were counted, from both the upper and the visceral sides, and their major axes were measured with a vernier calliper.

### *In vitro* and *in vivo* luciferase assays

Hepa 1–6 cells transfected with pCMV.lux and pCAG.lux plasmids were assayed for luciferase activity in a luminometer Revelation 4.28 (Dynex Technologies, Chantilly, VA). Light was measured for 4 s, starting 0.1 s after reagent addition, and the values were expressed as relative light units (R.L.U). Aliquots of the lysates were used to determine protein concentration, and R.L.U were normalized and expressed as R.L.U/mg protein. For bioluminescence live imaging, the method of Nguyen et al. [28] was used, with a Bioluminescence Optical Imager (IVIS 200; Xenogen, Alameda, CA).

### Histopathological studies

Samples of livers and lungs were fixed overnight in 10% buffered formalin, embedded in paraffin, sectioned 4 μm thick and stained with hematoxilin-eosin. Photographs were taken using an Olympus DP73 digital camera. Proliferating lesions were classified as hyperplasias and neoplastic lesions according to current histopathological criteria.

### Other microscopic techniques

For immunofluorescence analysis of proliferation, Hepa 1–6 cells were synchronized by serum starvation and transfected with pcDNA3/CAG-HA*Gas1* plasmid. Five hours after transfection, fresh FBS-containing medium was added. Fifteen hours later medium was replaced by fresh medium supplemented with FBS and 10μM BrdU, and cells were incubated at 37°C for 15 min. After incubation and washing, cells were stained with a mixture of anti-HA and anti-BrdU antibodies by standard methods and analyzed with a fluorescence microscope.

For X-gal staining of tissue samples after HGD, 10 μm frozen liver sections were fixed, air-dried, washed once with PBS and incubated at 37°C with pre-warmed X-gal solution until the blue staining was visible. Cells were counterstained with hematoxylin-eosin.

For immunofluorescence studies in the liver of transfected animals, livers were carefully extracted and left in cold phosphate buffer for 4 to 6 hours after 15 min perfusion with 4% paraformaldehyde in phosphate buffer (0.1M, pH 7.2). 100 μm thick vibratome sections were incubated with the primary antibody (1: 40) for 48 hours at 4°C and then with the secondary antibody (anti-goat Alexa 555, 1:500). All sections processed for immunofluorescence were counterstained with 4',6-diamidino-2-phenylindole (DAPI) and mounted on slides and coverslipped using Permafluor mounting medium (Immunon/Shandon, Pittsburgh, PA, USA). Then, the sections were either observed on a Zeiss standard epifluorescence microscope or under a confocal microscope (Leica, Wein, Austria; TCSSPE). Z-series of optical sections (1 μm apart) were obtained using sequential scanning mode. These stacks were processed with LSM 5 Image Browser software.

### Microarray study of gene expression

An Agilent 1 Color‐Gene Expression SurePrint G3 Mouse GE 8x60K Microarray Kit with Design ID 028005, containing 39,430 Entrez Gene RNAs and 16,251 lincRNA was used.100 ng of total RNA were labeled using LowInputQuick Amp Labeling kit (Agilent 5190–2305) following manufacturer instructions. Briefly, mRNA was reverse transcribed in the presence of T7-oligo-dT primer to produce cDNA, which was transcribed *in vitro* with T7 RNA polymerase in the presence of Cy3-CTP to produce labeled cRNA. The labeled cRNA was hybridized to the microarray according to the manufacturer's protocol. The arrays were washed, and scanned on an Agilent G2565CA microarray scanner at 100% PMT and 3 μm resolution. Intensity data was extracted using the Feature Extraction software (Agilent).

Raw data were taken from the Feature Extraction output files and were corrected for background noise using the normexp method [29]. To assure comparability across samples we used quantile normalization [30].

### Nucleic acids extraction, semi-quantitative and real time PCR

DNA was extracted from 100–150 mg liver portions in the presence of a protease inhibitor cocktail and purified after proteinase K digestion with phenol:chloroform.

RNA was extracted and purified from livers and from Hepa 1–6 cells with the Illustra RNAspin Mini RNA Isolation Kit (GE Healthcare Biosciences, Pittsburgh, PA), according to the manufacturer’s instructions. One μg of total RNA was retrotranscribed to cDNA using Superscript II RNase H^-^ (Invitrogen), following the manufacturer’s instructions, with random hexamers to prime the elongation reaction. Negative controls (no enzyme) were also included to check the possible contamination of the samples by genomic DNA. qRT-PCR was carried out in an ABI GeneAmp 7000 Sequence Detection System (Perkin-Elmer, Applied Biosystems, Foster City, CA) and analyzed with the ABI Prism Software (Applied Biosystems). The relative expression values were calculated as described by Pfaffl [31]. The primers used for semiquantitative PCR and qRT-PCR are given in the S1 Table.

### Protein extraction and western blot analysis

Fragments of liver (100 to 150 mg) were homogenized in 1ml of ice-cold PBS, supplemented with 2 μl of protease inhibitor cocktail (Roche). Total protein extraction was carried out with RIPA buffer (50mM Tris-HCl, 150mM NaCl, 1% Nonidet P-40, 0.5% sodium deoxycholate, 0.1% SDS, 2μl/ml protease inhibitor cocktail, pH 8) for 2 h at 4°C. The lysates were centrifuged at 14,000×*g* for 10 min at 4°C, the supernatants containing the soluble proteins were recovered and total protein was determined with the Bio-Rad protein assay reagent according to the manufacturer’s instructions. Western blots were developed after standard handling with the ECL advance detection kit (GE Healthcare, UK) according to the manufacturer’s instructions.

### Statistical analyses

Quantitative values were expressed as mean ± SD. Data in the different treatments of cells, groups of animals and RT-PCR determinations were compared by two-tailed t-test. In microarray experiments, statistical analyses and heatmaps were performed using R Bioconductor software (http://www.bioconductor.org/). Differentially expressed genes were identified with the multtest package [32].

## Results

### Overexpression of *Gas1* arrests proliferation of Hepa1-6 cells

It has long been established that *Gas1* overexpression arrests cell cycle before S phase in different cell lines [6], but its role in hepatoma cells remains unknown. Therefore, we analyzed the effects of *Gas1* overexpression in the Hepa 1–6 cell line proliferation.

**Fig 1:**
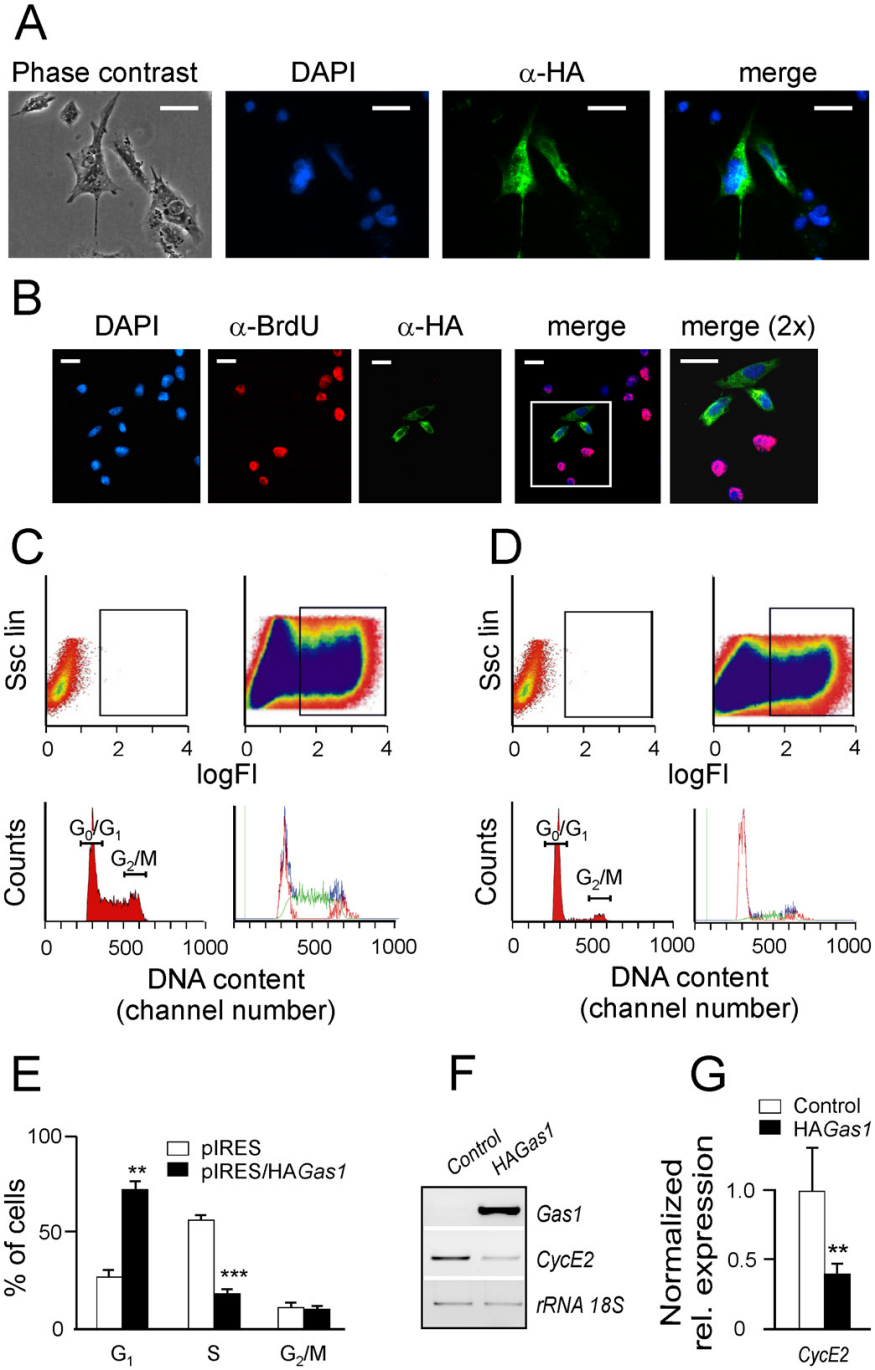
Transfection of Hepa 1–6 cells with the growth-arrest specific 1 (*Gas1*) gene. (A) Anti-HA immunofluorescence of Hepa 1–6 cells transfected with pcDNA3/CAG-HA*Gas1* in the absence of detergents to preserve the integrity of membranes. Nuclei were counterstained with DAPI. (B) Double immunofluorescent staining anti-HA/anti-BrdU of Hepa 1–6 cells transfected with pcDNA3/CAG-HA*Gas1*. (C) Cell cycle analysis of GFP-positive HEPA 1–6 cells after transfection with pIRES/GFP empty vector. 10^6^ cells (areas indicated in the upper row) were sorted and subjected to cell cycle analysis after propidium iodide staining (lower row). (D) As (C), after transfection with pIRES/HA*Gas1*. (E) Quantitation of the % of cells in the different stages of the cell cycle from the flow cytometry analysis. Experiments were done in triplicate. (F) Analysis of *Ccne2* expression in Hepa 1–6 cells overexpressing *Gas1*. A representative RT-PCR showing *Gas1* and *Ccne2* expression in cells transfected with either empty pcDNA3/CAG (control) or pcDNA3/CAG-HA*Gas1* (HAGas1), 15 h after transfection. (G) qRT-PCR to determine *CycE2* expression in cells as in (F). qRT-PCR was performed in triplicate from three independent experiments. Values were averaged and normalized to 18S *rRNA*. **, p<0.01. ***, p<0.001. In A and B the bar represents 11 μm.

In order to choose the strongest promoter to induce expression of *Gas1* in these mammalian cells, we compared the efficiency of the widely used CAG and CMV promoters by a luciferase assay. In this assay, using Hepa 1–6 cells routinely transfected with around 45% efficiency (S1A Fig), the CAG promoter drives an expression 14-fold higher than the CMV promoter (S1B Fig). Therefore, the former was used for the subsequent overexpression experiments. Endogenous GAS1 protein is not detectable in asynchronously growing Hepa 1–6 cells. However, an exogenously expressed HA-tagged GAS1 can be detected with both anti-HA and anti-GAS1 antibodies in these cells (S1C Fig), localizing to the cell membrane (Fig 1A).

These results show that the exogenous HA-*Gas1* can be differentially detected and that it is correctly incorporated into the cell membrane, like the endogenous counterpart. Further, we analyzed the effects of *Gas1* overexpression on cell proliferation. Asynchronously growing Hepa 1–6 cells transfected with pcDNA3/CAG-HA*Gas1* were unable to uptake BrdU as determined by double immunostaining against HA and BrdU, suggesting that cells overexpressing *Gas1* were unable to progress to the S phase of the cell cycle (Fig 1B).

To further analyze this effect, control and HA*Gas1*-expressing cells were sorted out by FACS and their cell cycle stage analyzed by propidium iodide staining (Fig 1C and 1D). The *Gas1*-overexpressing population was considerably enriched in G_0_/G_1_ cells, while the number of cells progressing to the cell cycle was highly reduced. Quantification of these results (Fig 1E) shows a significant (p < 0.001) reduction of S-phase cells in the *Gas1-* overexpressing populations (from 58.5 to 17.9%), correlating with a significant decreased expression of cyclin E2 (*Ccne2*) gene, known to be mandatory for cell cycle progression to S phase (Fig 1F and 1G). These results reinforce the anti-proliferating effects of GAS1 in Hepa 1–6 cells.

### Effects of *in vivo* overexpression of *Gas1* on the growth and malignancy of HCC

Given that *Gas1* overexpression arrests cell proliferation at the G_1_/S interface in the Hepa 1–6 hepatoma cell line, we wondered whether *Gas1* overexpression in liver could also affect proliferation of hepatoma cells *in vivo*. Firstly, HCC was induced by treating infant mice with DEN [33]. This carcinogen produces liver tumors as the only primary lesions, which later metastasize, mainly by the hematogenous route, to lungs [34]. When HCC was fully developed, mice were subjected to HGD to transfect liver cells with either a *Gas1*-overexpressing vector or an empty vector as control.

To determine the efficiency of the transfection by HGD, a construct carrying the *lacZ* reporter gene (pcDNA3.1/V5-His-TOPO/*lacZ*) was delivered in the conditions described under Materials and Methods and the β-galactosidase-positive cells visualized after X-gal staining (S2A Fig). In our hands, the transfection efficiency routinely lay between 30 and 40% and, as expected, most, if not all of the transfected cells, were in the neighborhood of blood vessels. These results are in accordance with published data [35]. We then checked whether the CAG promoter was also more efficient than the CMV promoter *in vivo*. Mice were transfected by HGD with plasmids carrying the luciferase gene under the control of either CMV (pCMV.lux) or CAG promoter (pCAG.lux). Forty eight hours later, live bioluminescent imaging showed that the CAG promoter is also the most efficient promoter *in vivo* (S2B Fig). The quantification of the light emitted in live animals after HGD (S2C Fig) revealed that the CAG promoter drives a luciferase activity more than 12-fold higher than the one induced by the CMV promoter.

**Fig 2:**
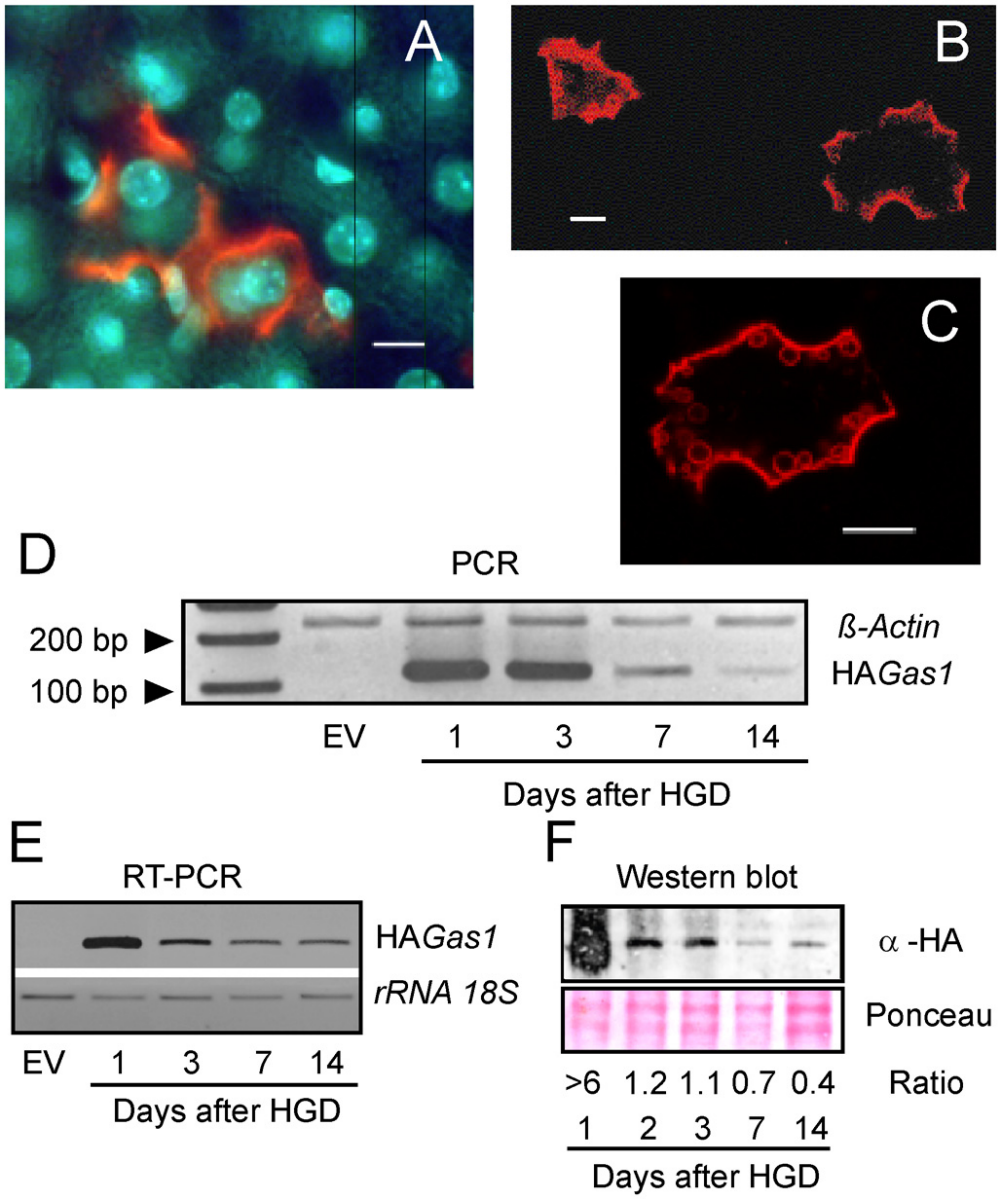
*In vivo* transfection of mice by HGD with pcDNA3/CAG-HA*Gas1*. (A) Immunofluorescence anti-HA on a liver section transfected with pcDNA3/CAG-HAGas1. Nuclei were counterstained with DAPI. (B) Confocal microscopy image of a liver section as that in (A). (C) A magnified confocal microscopy image as in B, to show the deposition of HA-tagged GAS1 into plasma membranes. (D) PCR using primers annealing to the HA tag of the *Gas1* construct at several times after HGD. As an internal control, a region of the constitutive β-actin gene was also amplified. (E) RT-PCR showing the HA-*Gas1* expression after HGD. 18S *rRNA* was used as loading control. EV, cells transfected with empty vector as control. (F) Western blot against the HA-tag to detect HA-GAS1 after HGD. The intensity of the anti-HA signal, scanned and normalized to the protein load as given by the Ponceau staining, is shown in the row marked “Ratio”.

We next checked whether the HA-GAS1 protein localizes to the plasma membranes after the *in vivo* transfections as it does *in vitro*. Fig 2A–2C shows that GAS1 is present and actively being assembled into plasma membranes in livers fixed 24 h after HGD, as evidenced by the presence of GAS1-carrying vesicles in a focal plane of a confocal microscope image (Fig 2C). Immunohistochemical analyses using an anti-GAS1 antibody showed that only the cells in the neighborhood of blood vessels are GAS1-positive, while the endogenous content of the protein is negligible in the remaining cells (results not shown).

The extent of the effects of *Gas1 in vivo* depends on the persistence of its product. Consequently, mice were transfected with pcDNA3/CAG-HA*Gas1* and transgene, mRNA and protein levels were analyzed over time. The persistence of the plasmid was checked by PCR after HGD, being high up to 72 h after HGD, but dropping to be almost negligible 14 days after HGD (Fig 2D). The concentration of mRNA follows a similar kinetics (Fig 2E) and protein levels are still high 14 days after HGD (Fig 2F).

On these grounds, animals developing DEN-induced HCC were transfected weekly by HGD with HA-*Gas1* to ensure steady levels of protein during the course of the experiment. The experiment was carried out in three groups of animals, 9 mice each. Animals from groups T (after tumor) and G (after *G* *as*1) received an intraperitoneal injection of DEN 15 days after birth, while those of the C (after control) group were injected with saline as a DEN-negative, control group. In a previous experiment, we detected that HCC was fully developed in DEN-treated animals between 30 and 35 weeks after birth. For this reason, HGD started in all groups at 28 weeks of age. Mice from group G were treated weekly with pcDNA3/CAG-HA*Gas1* until week 34 inclusive, while groups C and T were treated with the empty plasmid also administered by HGD. Thirty five weeks after birth, all the animals were sacrificed and their livers and lungs removed for analysis. The experiments were repeated twice. Histological studies were carried out in one of the experiments, but the PCR analysis of gene expression was repeated in both of them.

**Fig 3:**
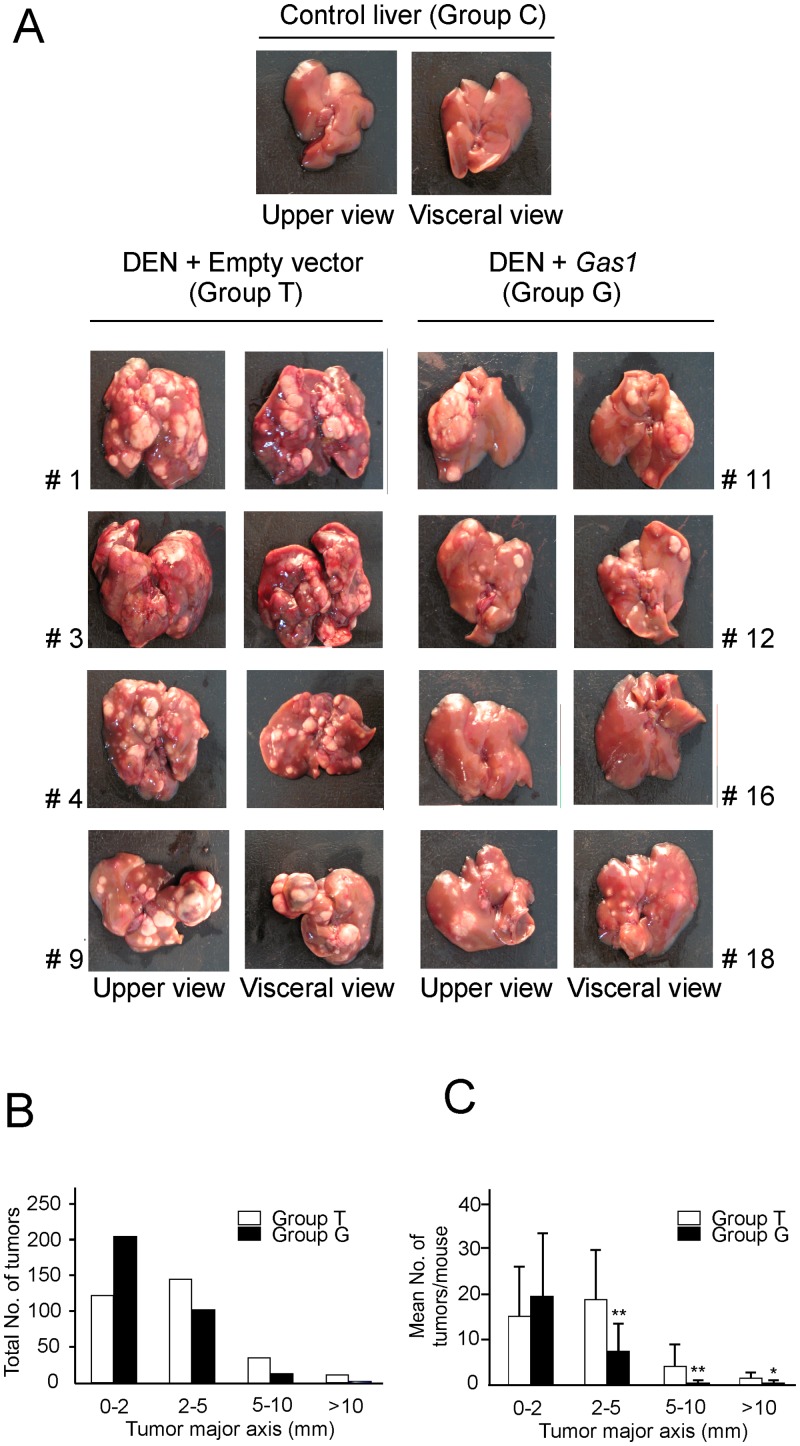
Effects of *Gas1* overexpression on the development of hepatocellular carcinoma. (A) Representative upper and visceral views of livers from animals of the three groups. (B) Total number of tumors present in the two major lobes of the livers of animals from groups T and G, arranged according to the length of their major axes. (C) Mean number of tumors per animal from groups T and G, arranged as in B. The bars represent the SD. **, p<0.05. *, p<0.1.

The *de visu* analysis of livers shows that all DEN-treated animals developed a large number of tumors; nevertheless, their size is reduced in animals overexpressing *Gas1*. The livers of 4 representative animals from groups T and G are shown in Fig 3A, together with that of a DEN-negative, control animal. The quantitative analysis of the data from all the animals is given in Fig 3B and 3C. The number of large tumors (>2 mm diameter) is significantly reduced following overexpression of *Gas1*, albeit the total number of lesions did not significantly differ between mice from groups T and G. In the 9 animals of group T a total of 319 tumors were counted, and 325 were found in those of group G (Fig 3B and 3C).

Histological studies were also carried out in the livers and lungs of all the animals. The analysis of livers allowed us to evaluate the grade of proliferating activity and malignancy of the different tumors. The sequential malignant transformation of hepatic cells after DEN treatment is shown, as an example, in S3 Fig. The examination of lungs was of interest because this organ is one of the main targets of HCC metastases [36] especially in DEN-induced carcinogenesis [34].

**Fig 4:**
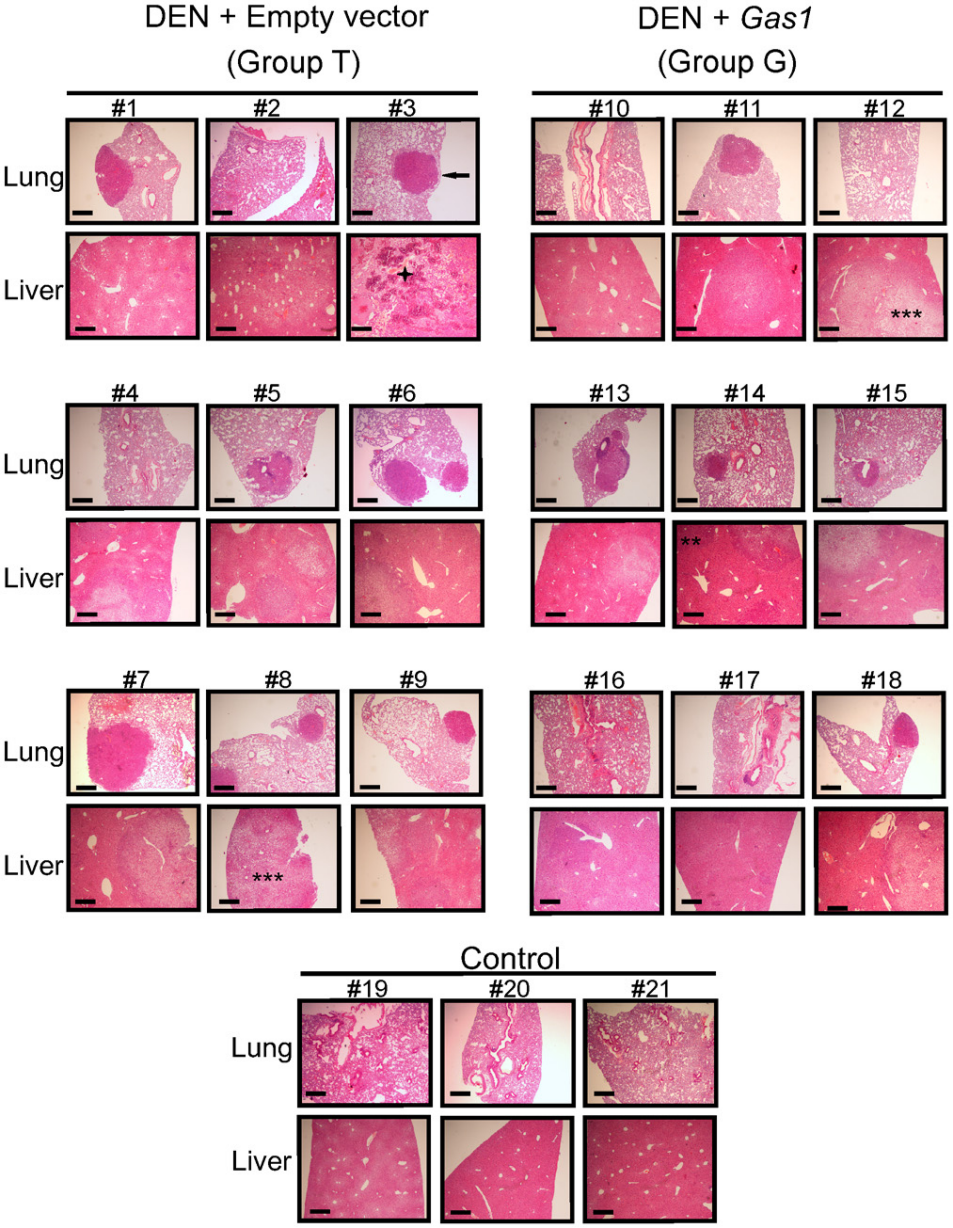
Histological analysis of livers and lungs. Representative sections of lungs and livers from all the animals of groups T and G and from 3 animals of group C are shown. As an example, the arrow points to a metastatic nodule in the lung of mouse No. 3. In the livers, examples of hyperplastic (*), adenoma (**), carcinoma (***), and necrotic (+) areas are indicated. The horizontal size bar corresponds to 200 μm. All the samples were stained with hematoxylin-eosin.

All the phases of malignant transformation, and even extensive necrotic areas were seen in the livers of the animals from group T (Fig 4). The livers of the animals transfected with *Gas1*, while developing DEN-induced HCC (group G) showed more normalized areas, although hyperplastic, adenomatous, and some carcinoma areas were still visible. As to the lungs, metastases were more frequent in animals from group T than in those from group G. The histological results referred to all the animals are summarized in Table 1, which shows that overexpression of *Gas1* after transfection by HGD is compatible with a reduction of the pathological manifestations of the HCC. The size of the sample does not allow an estimation of the significance of these results.

**Table 1:** Summary of the histological studies.

Pathological condition	Number of occurrences in DEN-treated animals transfected with	
	Empty plasmid (Group T)	*Gas 1* (Group G)
Hyperplasia	5/9 (56%)	7/9 (78%)
Adenoma	9/9 (100%)	7/9 (78%)
Carcinoma	9/9 (100%)	6/9 (67%)
Metastasis	7/9 (78%)	5/9 (56%)

### *In vivo* transfection of livers with *Gas1* partially restores the levels of gene expression

**Fig 5:**
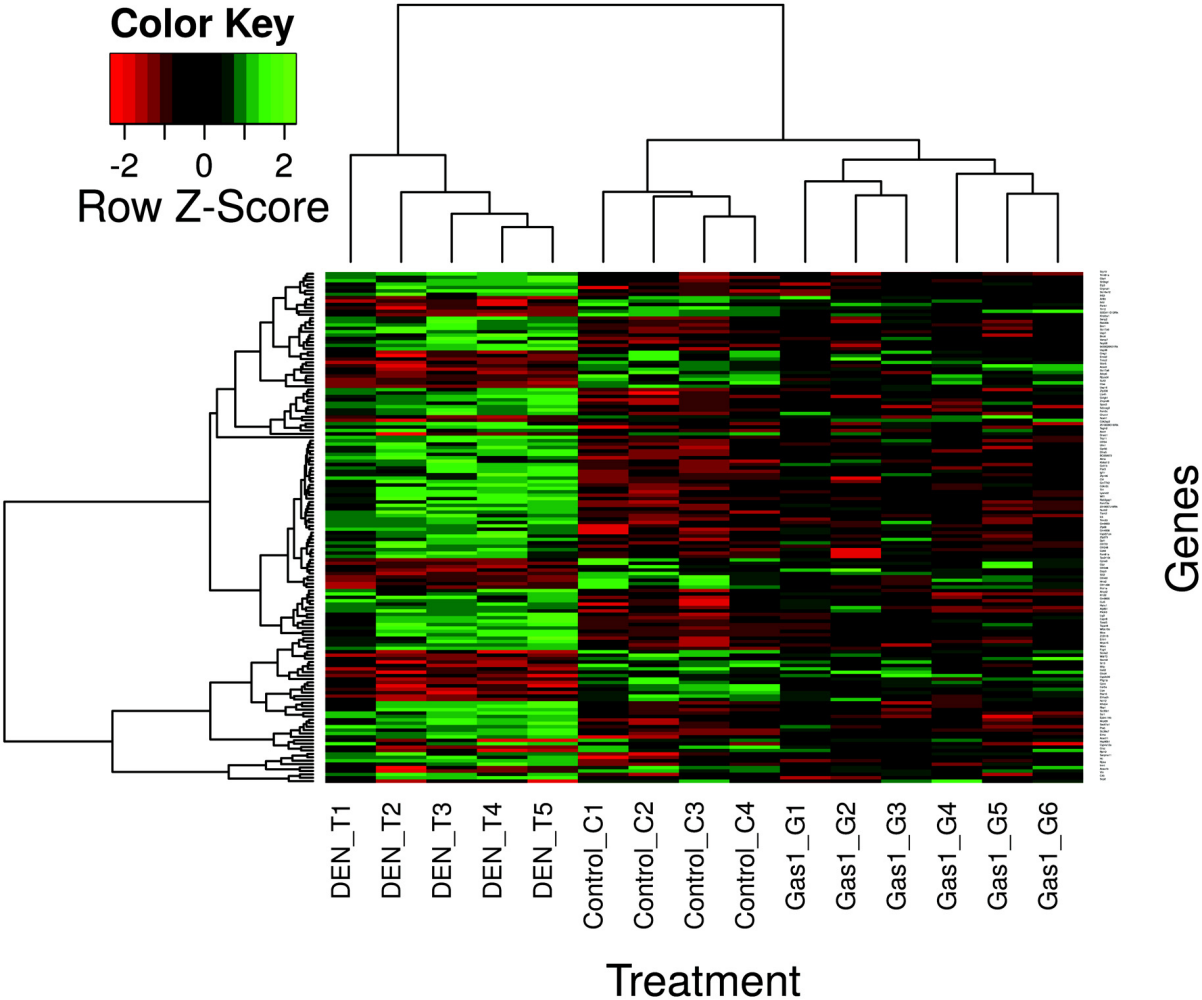
Effects of liver transfection with *Gas1* in restoring gene expression in DEN-treated animals. The heatmap shows the expression level of the genes up- or down-regulated in DEN-induced liver tumors, which reverts towards control levels (p>0.05) after transfection with *Gas1*.

To determine the effects on gene expression in tumor-bearing animals after *in vivo* transfection with *Gas1*, a microarray analysis was carried out with RNA from the livers of animals from the three groups described above. The raw data are accessible in the GEO database (GSE63574). By comparing the data from group C with those of group T, we found that the expression of 698 out of the 20758 genes analyzed was significantly affected (p<0.05) as a consequence of the DEN treatment. Then, the data from group T were compared with those of group G. In this way, we found that transfection of livers with *Gas1* significantly (p<0.05) affects the expression of 180 out of the above mentioned 698 genes (S4 Fig). Finally, the comparison of the expression level of these 180 genes with that of the same genes in the control animals revealed that the expression of 150 out of them was similar in groups G and C. These genes are listed in S2 Table. The results of this triangular study were summarized in S5 Fig. The heatmap (Fig 5) shows that mice treated with DEN and subjected to *Gas1* overexpression have a gene expression profile comparable to that of control, healthy mice, in contrast with those treated with DEN but transfected with an empty vector. This suggests that the gene expression profile in HCC-developing mice reverts towards physiological levels after *Gas1* overexpression.

**Fig 6:**
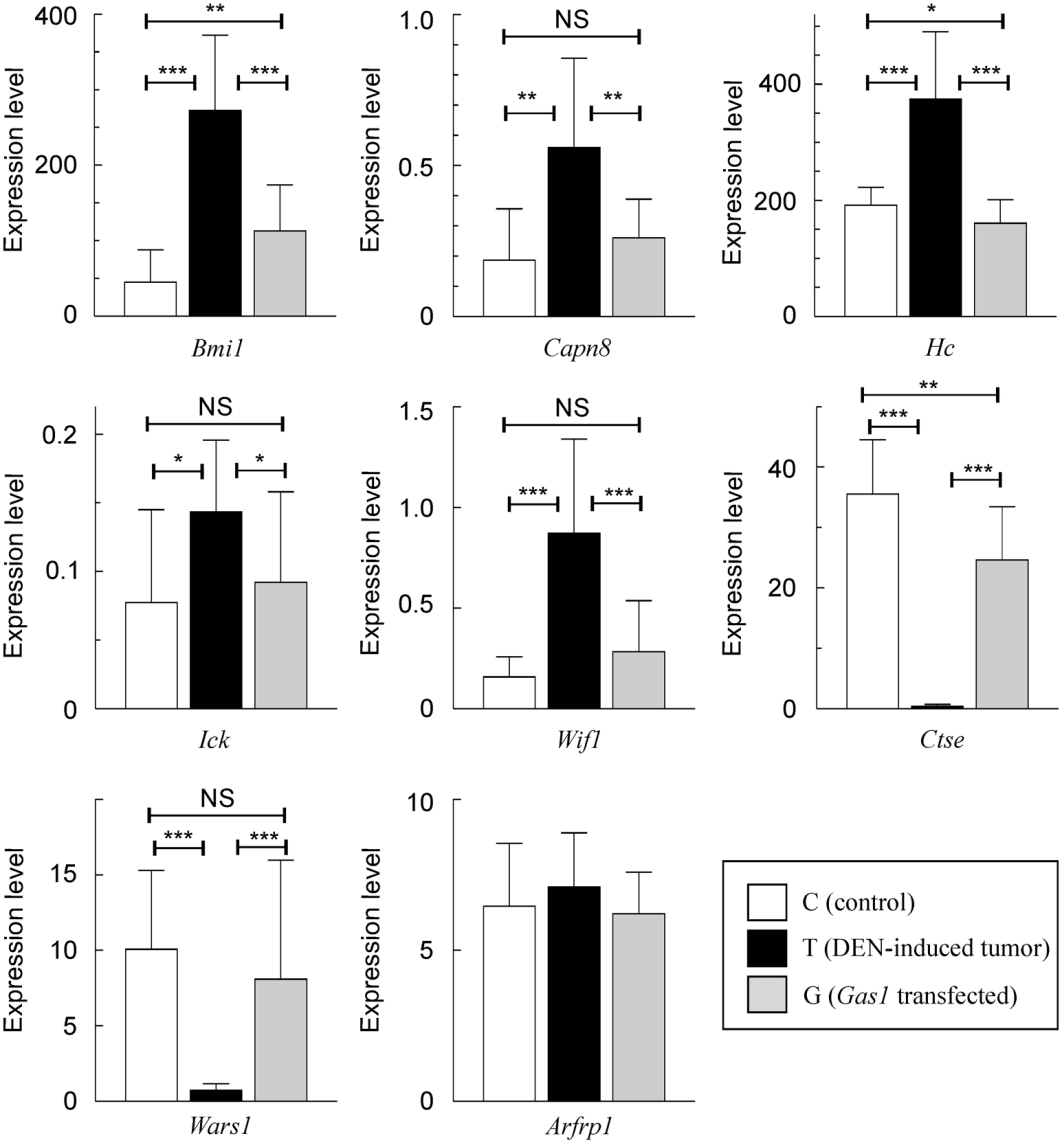
qRT-PCR validation of the expression of some of the genes shown in Fig 5. The analyses covered 5 genes up-regulated in tumors, 2 genes down-regulated and a control, unaffected gene. qRT-PCRs were run in triplicate and normalized relative to the β-actin expression for 4 control, 4 DEN-treated, and 6 DEN-treated plus *Gas1*-transfected animals as depicted in the lower-right inset. The values plotted are the mean ± SD of all the data. *, p<0.1. **, p<0.01. ***, p<0.001. NS non-significant.

The results obtained in the microarray analysis were validated by qRT-PCR for some genes of particular interest (Fig 6). These include five genes up-regulated, and two genes down-regulated in tumors. In all the instances, their expression level is restored after *Gas1* transfection. The qRT-PCR analysis of Fig 6 also includes, as a control, the expression of *Arfrp1*, one of the genes unaffected by DEN treatment.

## Discussion

In this study we used *in vitro* and *in vivo* murine models to understand the effects of *Gas1* overexpression on the development and metastatic potential of HCC. Transfection of Hepa 1–6 cells and live mice with HA-tagged *Gas1* under the control of the CAG promoter resulted in the proper expression of the gene and in the correct localization of its product to the plasma membrane (Figs 1A and 2). Overexpression of *Gas1* resulted in the inhibition of the proliferation of Hepa 1–6 cells, which did not progress to S phase (Fig 1E). Interestingly, the *Gas1*-induced cell cycle arrest is accompanied by the inhibition of *Ccne2* transcription (Fig 1G), although the present results do not allow us to establish a causal relationship between both phenomena. However, hepatoma cells add to other cell lines in sharing this effect of *Gas1* overexpression (see, for instance refs. [6,10,11,37,38].

Most importantly, the present study shows that the antiproliferating effects of *Gas1* on hepatoma cells can be extrapolated *in vivo*. DEN-induced HCC in mice was used as a model system, and livers were transfected by HGD to overexpress Gas1. The maximum reported efficiency of this procedure is 50% [39], although the normal values rank from 10 to 40% [35]. In our hands, values between 30 and 40% were systematically obtained. Although these values were obtained with a construct carrying the *lacZ* gene (pcDNA3.1/V5-His-TOPO/*lacZ*), the similarity in size of this plasmid with the one used to overexpress *Gas1* (pcDNA3/CAG-HA*Gas1*) allowed us to assume that the transfection efficiency is alike. Despite the fact that roughly two thirds of the liver cells did not overexpress *Gas1*, a noticeable reduction in the size of hepatoma foci was achieved, as shown in Fig 4. It has been described that overexpression of *Gas1* is able to reduce the tumor volume due to both, inhibition of cell proliferation and induction of apoptosis [11,18,40]. Further studies are needed to clarify whether either or both of these causes contribute to decrease tumor size in liver.

Histological studies facilitate the objective determination of the transformation grade of the liver tumors and, taking into account that lung is a primary target organ for metastatic colonization by HCC cells, the lungs of all the animals were also analyzed for metastatic foci. The results summarized in Table 1 show an excellent agreement with those given in Figs 4 and 5 in that, for instance, *Gas1* overexpression reduces the number of highly malignant lesions, while increases the number of just hyperplastic areas.

In addition to the *de visu* examination, we also analyzed the global gene expression in the livers of animals from the three groups described above, namely, control animals (C), DEN-treated, tumor-bearing mice (T) and tumor-bearing mice after liver transfection with *Gas1* (G). The results of the microarray analysis revealed that the expression level of 150 genes is restored after *Gas1* transfection (Fig 5). The expression changes in seven out of these 150 genes have been checked by qRT-PCR (Fig 6). Some of them have a special oncologic interest. For instance, *Bmi1* codes for a polycomb gene repressor and is overexpressed in human HCC. Interestingly, knockdown of *Bmi1* inhibits cancer cell growth *in vivo* [41], and it has been recently proposed that this gene may be a potential target for innovative treatments against human liver cancer [42]. Of note, *Bmi1* is clearly overexpressed in DEN-induced liver tumors, but transfection with *Gas1* restores its expression level. The expression of *Ctse* dropped to negligible levels in the liver of DEN-treated animals, an effect that reverts after *Gas1* transfection. Interestingly, cathepsin 8 induces growth arrest and apoptosis in tumor cells, but not in normal cells [43]. A similar effect of *Gas1* transfection was observed in the expression of *Wars1*, a gene whose low expression has been correlated with poor prognostic in colorectal cancer patients [44].

Thirteen out of the 150 genes whose expression level is restored after *Gas1* transfection, namely *Bmi1*, *Sulf1*, *Cd40*, *Ift122*, *Wif1*, *Igf1r*, *Rbp1*, *Cul5*, *Akna*, *Ick*, *Cdkn2c*, *Ift52* and *Slit2* are related in some way to the hedgehog signaling [45–64]. These genes include some of those analyzed by qPCR in Fig 6 (*Bmi1*, *Wif1* and *Ick*). As the hedgehog pathway has been implicated in the development of HCC and of colonic cancer stem cells, as well as in the epithelial-mesenchymal transition (for reviews, see [65, 66]), the present results support the idea that the tumor-suppressing activity of GAS1 may be mechanistically related to its participation in hedgehog signaling.

Gobeil *et al*. [67] have reported, after a genome-wide shRNA screening analysis, that *Gas1* meets the criteria to be considered a melanoma metastasis suppressor gene. Specifically, a decrease in the expression of the gene increases the metastatic potential of melanoma cells, while does not affect the primary tumor growth. Our results clearly show that *Gas1* negatively affects the progression of HCC, but we do not yet know whether the reduction in the number of lung metastasis is simply a consequence of the action of GAS1 on primary liver tumors or it may also imply a metastasis suppressor role.

As far as we know, no reports have appeared in the literature concerning the changes in the levels of *GAS1* expression in human liver between healthy individuals and patients from HCC or other liver pathologies. These data will be very valuable to explore the potential of this gene for both prognostic studies and therapeutic protocols. Moreover, further research is required to explore the mechanisms by which this gene plays a tumor suppressor role. However, the present results are interesting and novel because they show for the first time that this role can be applied *in vivo* to a common and highly malignant neoplasia as HCC. Although the tumor suppressor effects of *GAS1* had been previously reported in cell cultures or in xenograft models, this is the first work in which the suppressor activity of murine *Gas1* is reported for primary tumors *in vivo*. Recent advances in the design of safe vectors for transgene delivery (reviewed in [68]) may result in extrapolating our results to humans and so a promising field of research emerges in the area of hepatic, neoplastic diseases.

## Supporting Information

S1 Fig Transfection of Hepa 1–6 cells. (A) Flow cytometry analysis of Hepa 1–6 cells after transfection with pIRES GFP/HA plasmid. On the left, signal from control, non-transfected cells; on the right, GFP fluorescent emission after transfection. (B) Luciferase assay in Hepa 1–6 cells transfected with pCMV.lux and pCAG.lux plasmids. Three independent transfections were performed in triplicate and the 9 values of relative luciferase units (R.L.U.) were normalized to total protein and averaged. Bars mean SD. (C) Western blot to detect the product of using anti-GAS1 and anti-HA antibodies.
(PDF)

S2 Fig Transfection of livers by HGD. (A) Efficiency of HGD. Mice were subjected to HGD with either empty vector or with a β-galactosidase-expressing plasmid. Frozen sections from the left lateral lobes were subjected to X-Gal staining, counterstained with hematoxylin and analyzed to estimate the transfection efficiency. (B) Bioluminescent live imaging of animals subjected to HGD with vectors expressing the luciferase gene under the control of either the CMV or CAG promoters. As a control, a group of animals was subjected to HGD with an empty vector. (C) Photon emission quantification of the animals shown in (B).
(PDF)

S3 Fig Progression of a normal liver towards HCC after DEN treatment. Hematoxylin-eosin stained sections of livers from normal and DEN-treated mice are shown. The magnification is indicated by the bars: top row, 125 μm; middle row, 50 μm; bottom row, 12.5 μm.
(PDF)

S4 Fig Effects of liver transfection with *Gas1* on gene expression. The heatmap shows the expression level of the genes up- or downregulated in DEN-induced liver tumors, whose expression changes significantly (adjusted p<0.05) after transfection with *Gas1*.
(PDF)

S5 Fig Summary of the triangular study analyzing the results of the microarray experiment. The expression level of the 20,758 genes of the microarray in control animals (Group C) was first compared with that of the DEN-treated, tumor-bearing animals (Group T) to find that the expression of 698 genes had significantly (p<0.05) changed. Then the expression level of these 698 genes was compared with that found in animals from group G (transfected with *Gas1*), and a significant change (p<0.05) in the expression of 180 genes was detected. Finally, the expression level of these 180 genes was compared with that in control animals to find that in 150 genes the levels are similar in both groups G and C (p≥0.05).
(PDF)

S1 Table Primers used for PCR. (DOCX)

S2 Table Genes whose expression is restored in livers of DEN-treated animals after *Gas1* overexpression. The 150 genes whose expression is restored after liver transfection with *Gas1* list shows the comparison of the expression of 150 genes with that of the control animals.
(DOCX)
