# Supplementary figures and images for: Growth Arrest Specific 1 (Gas1) Gene Overexpression in Liver Reduces the In Vivo Progression of Murine Hepatocellular Carcinoma and Partially Restores Gene Expression Levels
Source: PLoS One. 2015 Jul 10;10(7):e0132477. doi: 10.1371/journal.pone.0132477 (PMC4498802; doi:10.1371/journal.pone.0132477)

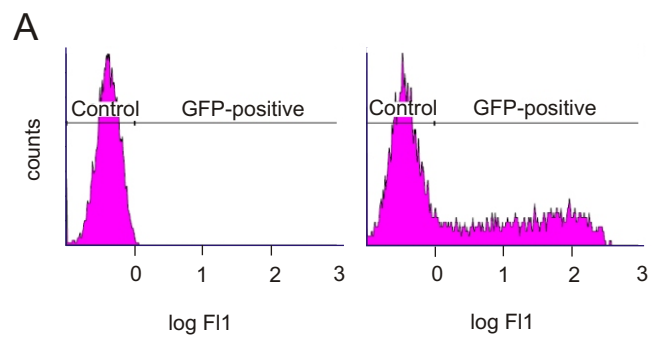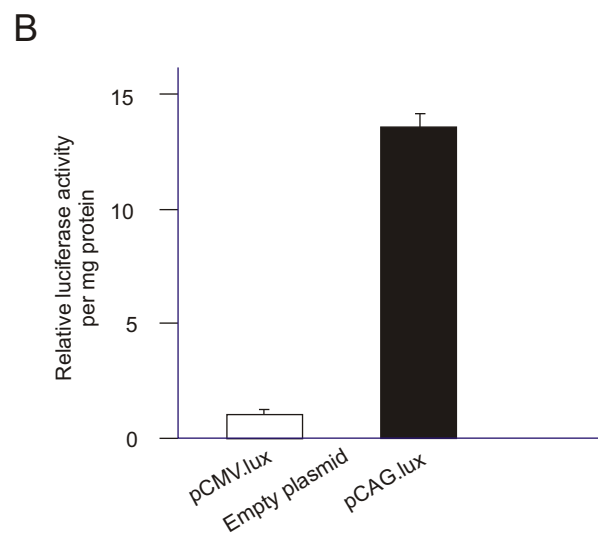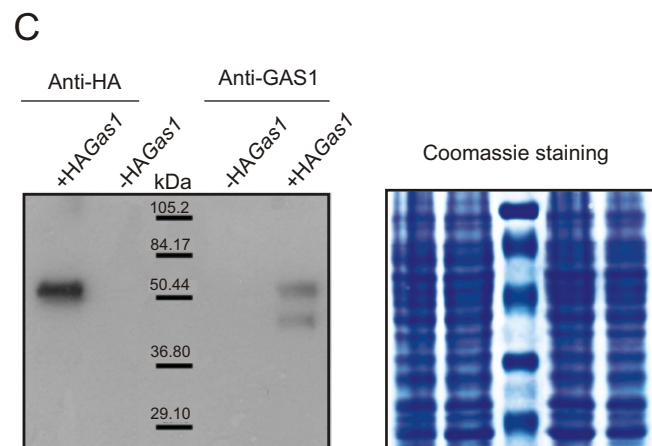

Supplement: S1 Fig — (A) Flow cytometry analysis of Hepa 1–6 cells after transfection with pIRES GFP/HA plasmid. On the left, signal from control, non-transfected cells; on the right, GFP fluorescent emission after transfection. (B) Luciferase assay in Hepa 1–6 cells transfected with pCMV.lux and pCAG.lux plasmids. Three independent transfections were performed in triplicate and the 9 values of relative luciferase units (R.L.U.) were normalized to total protein and averaged. Bars mean SD. (C) Western blot to detect the product of using anti-GAS1 and anti-HA antibodies. (PDF) [file pone.0132477.s001.pdf]

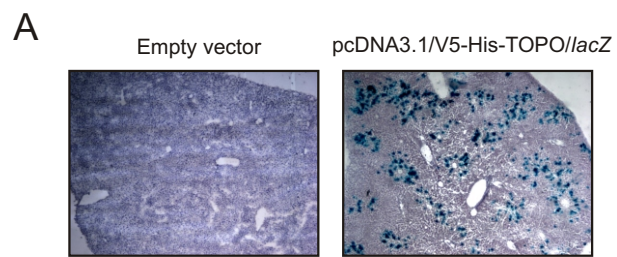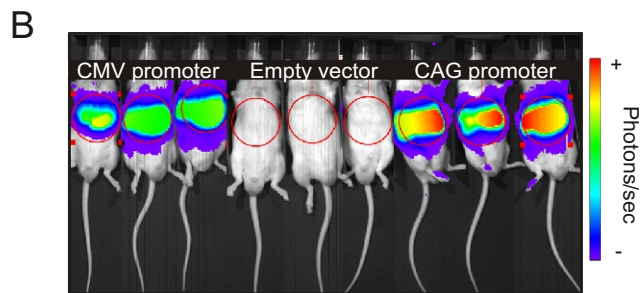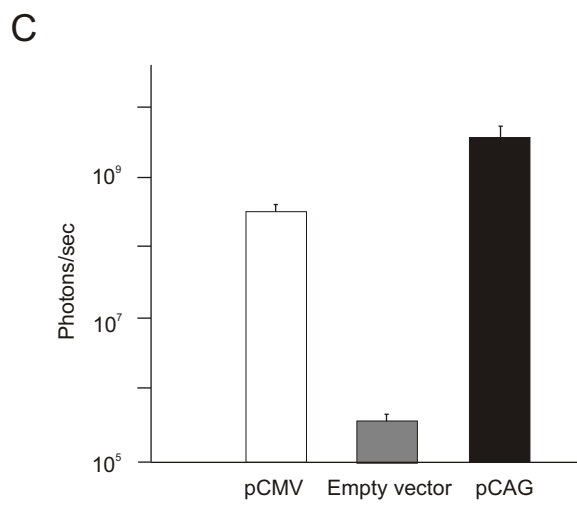

Supplement: S2 Fig — (A) Efficiency of HGD. Mice were subjected to HGD with either empty vector or with a β-galactosidase-expressing plasmid. Frozen sections from the left lateral lobes were subjected to X-Gal staining, counterstained with hematoxylin and analyzed to estimate the transfection efficiency. (B) Bioluminescent live imaging of animals subjected to HGD with vectors expressing the luciferase gene under the control of either the CMV or CAG promoters. As a control, a group of animals was subjected to HGD with an empty vector. (C) Photon emission quantification of the animals shown in (B). (PDF) [file pone.0132477.s002.pdf]

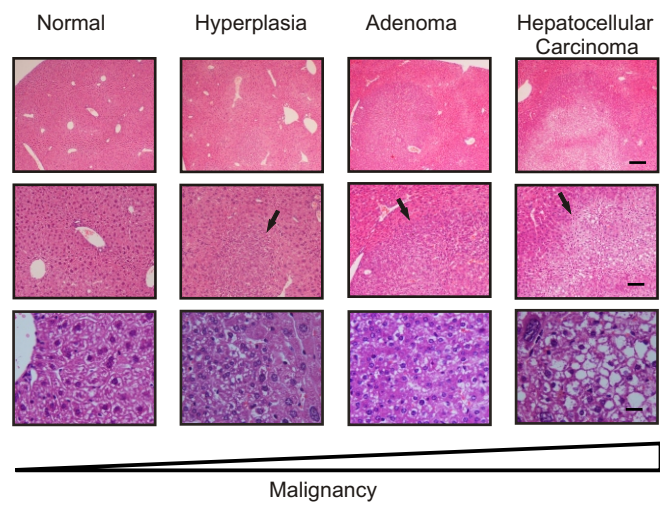

Supplement: S3 Fig — Hematoxylin-eosin stained sections of livers from normal and DEN-treated mice are shown. The magnification is indicated by the bars: top row, 125 μm; middle row, 50 μm; bottom row, 12.5 μm. (PDF) [file pone.0132477.s003.pdf]

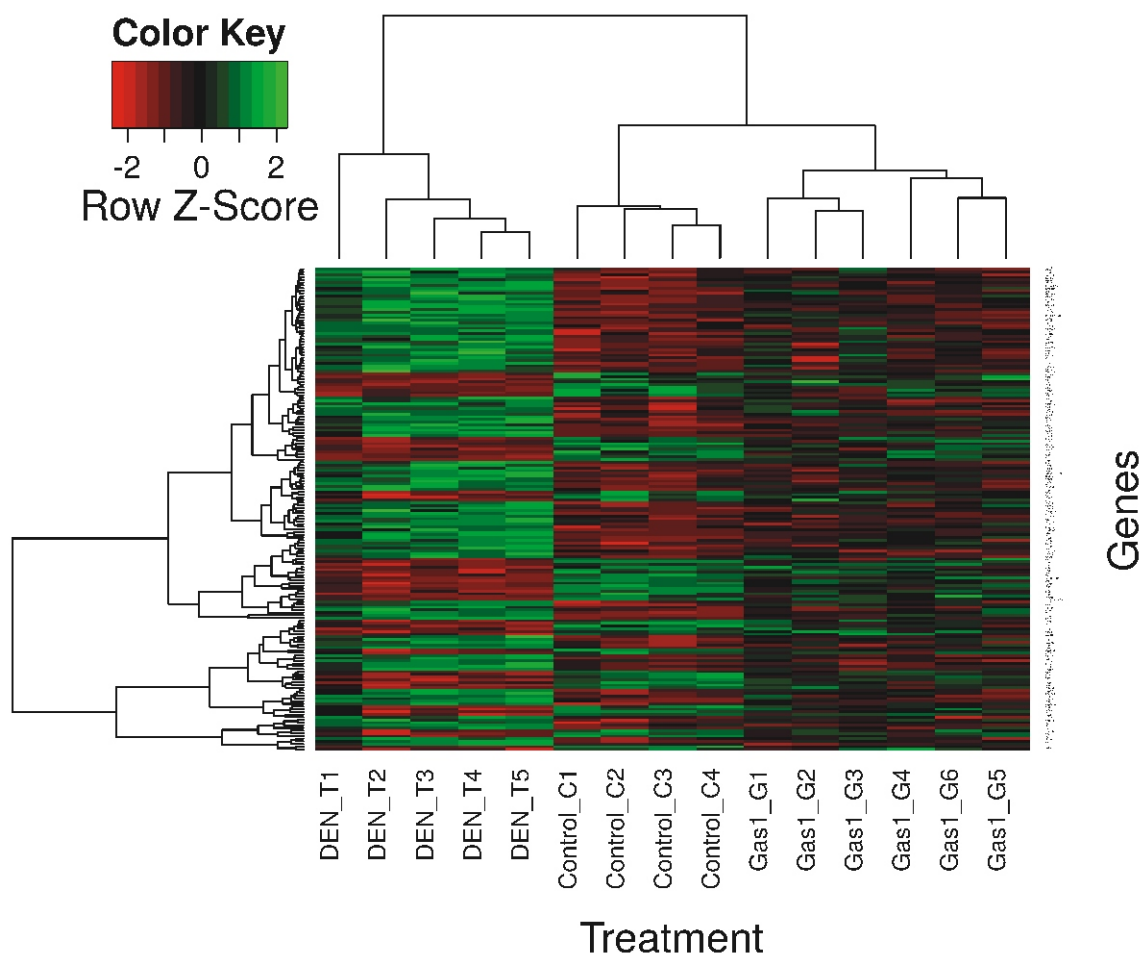

Supplement: S4 Fig — The heatmap shows the expression level of the genes up- or downregulated in DEN-induced liver tumors, whose expression changes significantly (adjusted p<0.05) after transfection with Gas1. (PDF) [file pone.0132477.s004.pdf]

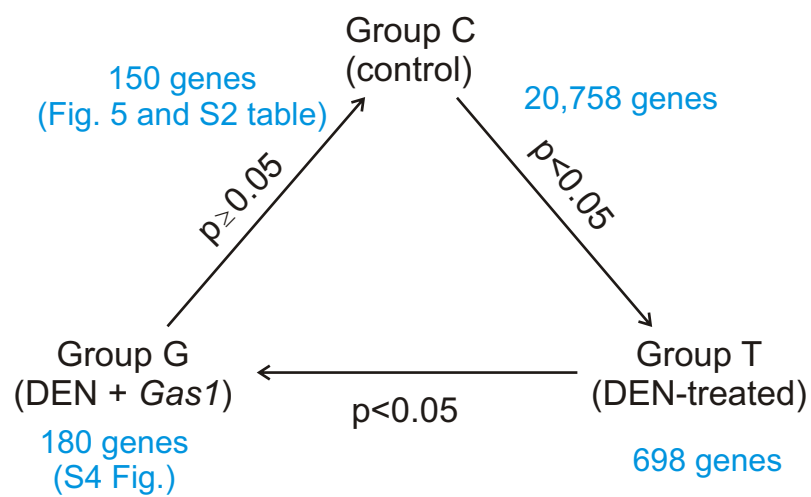

Supplement: S5 Fig — The expression level of the 20,758 genes of the microarray in control animals (Group C) was first compared with that of the DEN-treated, tumor-bearing animals (Group T) to find that the expression of 698 genes had significantly (p<0.05) changed. Then the expression level of these 698 genes was compared with that found in animals from group G (transfected with Gas1), and a significant change (p<0.05) in the expression of 180 genes was detected. Finally, the expression level of these 180 genes was compared with that in control animals to find that in 150 genes the levels are similar in both groups G and C (p≥0.05). (PDF) [file pone.0132477.s005.pdf]
