# Supplementary material for: Growth Arrest Specific 1 (Gas1) Gene Overexpression in Liver Reduces the In Vivo Progression of Murine Hepatocellular Carcinoma and Partially Restores Gene Expression Levels
Source: PLoS One. 2015 Jul 10;10(7):e0132477. doi: 10.1371/journal.pone.0132477 (PMC4498802; doi:10.1371/journal.pone.0132477)
Supplement: S1 Table — (DOCX) [file pone.0132477.s006.docx]

**Supplementary table I. Primers used for PCR**

| Gene | | Primer sequence | |
| --- | --- | --- | --- |
|  |  | forward reverse | |
| **Semiquantitative PCR** | | | |
| *HAtag-Gas1* | ATGGCCTACCCCTACGACGTG | | TCGCACACGCAGTCGTTGAG |
| *18S rRNA* | TGGTTGATCCTGCCAGTAGC | | CTCTCCGGAATCGAACCCTG |
| *CycE2* | TCTGGCGGTGAGGAGAGGTTTC | | ATGCAAGGGCTGATTCCTCCAG |
| **RT-PCR** | | | |
| *CycE2* | TTGGAATGGGATGACATCTCG | | AACAAAAGGCACCATCCAGTCT |
| *18S rRNA* | CACGGCCGGTACAGTGAAA | | AGAGGAGCGAGCACCAA |
| *Ctse* | AGGAGTGACCCCAGTGTTTG | | CATAGCCTCCGAAAGTCAGC |
| *Wars1* | TACTCAAGGGGAGCTCGTGA | | GTGGCTGAGGTGCATGTTTC |
| *Hc* | GAAGCAGGAAACCATTGGAA | | GCTCACCACCCCTTGTTTTA |
| *Wif1* | AGGCGAGAACTTCACAAGCA | | CTCTTCTCCGAGCCATGGTG |
| *Capn8* | AGGACTTCGAGACCCTGAGAA | | GGTCCAAGATCCCTGTAGCC |
| *Bmi1* | GCCTTCACTGTAACGTCTGGA | | ACTGAGACTTGGAGGGGTCA |
| *Ick* | GTGACTGTGCAGTTTAGTCGC | | GACAGGCTGATTGACCCCAA |
| *Arfrp1* | TGCAGTCTTTGTGGGACAAG | | CAGTGCTTCACTCGAAACCA |
| *β-actin* | GCTACAGCTTCACCACCACA | | TCTCCAGGGAGGAAGAGGAT |
