# Supplementary material for: Growth Arrest Specific 1 (Gas1) Gene Overexpression in Liver Reduces the In Vivo Progression of Murine Hepatocellular Carcinoma and Partially Restores Gene Expression Levels
Source: PLoS One. 2015 Jul 10;10(7):e0132477. doi: 10.1371/journal.pone.0132477 (PMC4498802; doi:10.1371/journal.pone.0132477)
Supplement: S2 Table — The 150 genes whose expression is restored after liver transfection with Gas1 list shows the comparison of the expression of 150 genes with that of the control animals. (DOCX) [file pone.0132477.s007.docx]

**Supplementary table II. Genes whose expression is restored in livers of DEN-treated animals after *Gas1* overexpression. The list shows the results of the comparison of the expression with that of the control animals.**

| **Row names** | **adjusted p** | **q** |
| --- | --- | --- |
| Cryz | 0.2609470 | 0.05309282 |
| Tmtc2 | 0.4236937 | 0.08620560 |
| Fam3c | 0.5159250 | 0.10497117 |
| Slc17a8 | 0.2585206 | 0.05259913 |
| Creg1 | 0.1757266 | 0.03575370 |
| Rpsa | 0.1757266 | 0.03575370 |
| Dpf1 | 0.4742114 | 0.09648403 |
| Scp2 | 0.4948183 | 0.10067677 |
| Capn8 | 0.3847712 | 0.07828635 |
| Zmynd8 | 0.2247366 | 0.04572539 |
| Zfp105 | 0.2585206 | 0.05259913 |
| Sfxn5 | 0.7197789 | 0.14644771 |
| Bmi1 | 0.4742114 | 0.09648403 |
| Churc1 | 0.8766798 | 0.17837109 |
| Hsp90b1 | 0.9137310 | 0.18590961 |
| Adcy6 | 0.2813085 | 0.05723562 |
| Olfr348 | 0.7331636 | 0.14917100 |
| Tiam2 | 0.5256194 | 0.10694361 |
| Tlr12 | 0.2913858 | 0.05928596 |
| Ffar3 | 0.1757266 | 0.03575370 |
| Vtn | 0.4198469 | 0.08542293 |
| Golgb1 | 0.1757266 | 0.03575370 |
| Nceh1 | 0.5256194 | 0.10694361 |
| Ahcyl2 | 0.9077225 | 0.18468710 |
| Sulf2 | 0.5180591 | 0.10540538 |
| Uspl1 | 0.9033047 | 0.18378824 |
| 2310057J18Rik | 0.7230022 | 0.14710353 |
| Gfpt1 | 0.4948183 | 0.10067677 |
| Trip11 | 0.9364430 | 0.19053064 |
| Cd40 | 0.4287233 | 0.08722894 |
| Srp19 | 0.4742114 | 0.09648403 |
| Ift122 | 0.3386679 | 0.06890608 |
| Golt1b | 0.4431434 | 0.09016287 |
| Acot1 | 0.1951884 | 0.03971345 |
| Dfna5 | 0.4236937 | 0.08620560 |
| Dnaic1 | 0.1757266 | 0.03575370 |
| Glod4 | 0.8957701 | 0.18225525 |
| Kbtbd12 | 0.3613726 | 0.07352561 |
| Olfr481 | 0.4146880 | 0.08437329 |
| Ehhadh | 0.4948183 | 0.10067677 |
| Acad11 | 0.2247366 | 0.04572539 |
| Preb | 0.2542108 | 0.05172226 |
| Wif1 | 0.2343419 | 0.04767970 |
| Zfp273 | 0.4475950 | 0.09106861 |
| Gstt2 | 0.7618617 | 0.15500996 |
| Atp8b1 | 0.5327094 | 0.10838616 |
| Igf1r | 0.2247366 | 0.04572539 |
| Enox2 | 0.5690925 | 0.11578874 |
| Klhdc4 | 0.7967132 | 0.16210093 |
| Tas2r104 | 0.2247366 | 0.04572539 |
| Olfr94 | 0.2247366 | 0.04572539 |
| Rbp1 | 0.4235273 | 0.08617175 |
| Cul5 | 0.1757266 | 0.03575370 |
| Sh3bgrl | 0.4896941 | 0.09963418 |
| Usp16 | 0.3960304 | 0.08057716 |
| Elp3 | 0.2839952 | 0.05778225 |
| Lig3 | 0.1757266 | 0.03575370 |
| Cdk2ap2 | 0.5975980 | 0.12158853 |
| Zfp68 | 0.1757266 | 0.03575370 |
| Cpne9 | 0.7530648 | 0.15322014 |
| Slc17a9 | 0.5256194 | 0.10694361 |
| Spcs3 | 0.6339968 | 0.12899430 |
| Fcgrt | 0.1757266 | 0.03575370 |
| Khdrbs1 | 0.2585206 | 0.05259913 |
| Usp38 | 0.2247366 | 0.04572539 |
| Olfr1490 | 0.2813085 | 0.05723562 |
| Erlin1 | 0.3960304 | 0.08057716 |
| Hyou1 | 0.7008361 | 0.14259356 |
| 9430020K01Rik | 0.2467919 | 0.05021279 |
| Rpusd4 | 0.2798761 | 0.05694418 |
| Lta4h | 0.4235273 | 0.08617175 |
| Olfr701 | 0.3960304 | 0.08057716 |
| Cyp2j7-ps | 0.4567279 | 0.09292680 |
| Hc | 0.4236937 | 0.08620560 |
| Enho | 0.1757266 | 0.03575370 |
| Ufm1 | 0.3960304 | 0.08057716 |
| Zc3h18 | 0.2224822 | 0.04526670 |
| Gipr | 0.6409743 | 0.13041396 |
| Tagln2 | 0.4948183 | 0.10067677 |
| Gm4858 | 0.2247366 | 0.04572539 |
| Cyp2c39 | 0.4742114 | 0.09648403 |
| Pik3r3 | 0.2839952 | 0.05778225 |
| Serpina11 | 0.1757266 | 0.03575370 |
| Trmt61a | 0.2247366 | 0.04572539 |
| Rpl12 | 0.1757266 | 0.03575370 |
| Ilvbl | 0.5256194 | 0.10694361 |
| Hmx2 | 0.1757266 | 0.03575370 |
| Polr1e | 0.2247366 | 0.04572539 |
| Sdccag3 | 0.2343419 | 0.04767970 |
| Inmt | 0.1757266 | 0.03575370 |
| Akna | 0.2247366 | 0.04572539 |
| BC050972 | 0.2913858 | 0.05928596 |
| Ick | 0.8844678 | 0.17995566 |
| 2510039O18Rik | 0.2467919 | 0.05021279 |
| Nol12 | 0.4475950 | 0.09106861 |
| C4b | 0.6409743 | 0.13041396 |
| Ctrl | 0.2599802 | 0.05289610 |
| Zfp330 | 0.1757266 | 0.03575370 |
| Fam73a | 0.2247366 | 0.04572539 |
| Tceal5 | 0.2247366 | 0.04572539 |
| Acaa1b | 0.2247366 | 0.04572539 |
| Car5a | 0.2247366 | 0.04572539 |
| Gpr56 | 0.2148271 | 0.04370917 |
| Arl8b | 0.1951884 | 0.03971345 |
| Gm7742 | 0.2229640 | 0.04536471 |
| Cdkn2c | 0.2343419 | 0.04767970 |
| Gnpnat1 | 0.3386679 | 0.06890608 |
| Vamp7 | 0.2542108 | 0.05172226 |
| Rab3gap1 | 0.2839952 | 0.05778225 |
| Wfdc15b | 0.2542108 | 0.05172226 |
| Tspan8 | 0.1757266 | 0.03575370 |
| Slc16a12 | 0.3141212 | 0.06391174 |
| Nucb2 | 0.2247366 | 0.04572539 |
| Mrps15 | 0.4475950 | 0.09106861 |
| Rab33b | 0.5849051 | 0.11900601 |
| Fam81a | 0.4742114 | 0.09648403 |
| Gm9855 | 0.2959356 | 0.06021168 |
| Wars | 0.3470028 | 0.07060191 |
| Senp2 | 0.7208333 | 0.14666225 |
| Pttg1ip | 0.2247366 | 0.04572539 |
| Miox | 0.1757266 | 0.03575370 |
| Trim33 | 0.6409743 | 0.13041396 |
| Mrpl20 | 0.1951884 | 0.03971345 |
| Wdr73 | 0.7024648 | 0.14292494 |
| Mttp | 0.2343419 | 0.04767970 |
| Dzip3 | 0.2343419 | 0.04767970 |
| Cpox | 0.1757266 | 0.03575370 |
| Ift52 | 0.3384038 | 0.06885233 |
| Acss3 | 0.2585206 | 0.05259913 |
| Pex16 | 0.1757266 | 0.03575370 |
| Ssr1 | 0.2229640 | 0.04536471 |
| Nop58 | 0.6645825 | 0.13521734 |
| Sec61a1 | 0.5690925 | 0.11578874 |
| Birc6 | 0.2839952 | 0.05778225 |
| Cyp4a12a | 0.1757266 | 0.03575370 |
| Epb4.1l4b | 0.2229640 | 0.04536471 |
| St13 | 0.2247366 | 0.04572539 |
| Gm9933 | 0.2542108 | 0.05172226 |
| Slit2 | 0.1951884 | 0.03971345 |
| Olfr248 | 0.8337182 | 0.16963003 |
| Slc39a7 | 0.3960304 | 0.08057716 |
| Pank1 | 0.1757266 | 0.03575370 |
| Lysmd2 | 0.1951884 | 0.03971345 |
| Tlr1 | 0.4475950 | 0.09106861 |
| Stoml2 | 0.3758201 | 0.07646514 |
| Ctse | 0.1757266 | 0.03575370 |
| Lipa | 0.3028782 | 0.06162423 |
| Slc4a2 | 0.2247366 | 0.04572539 |
| Slc35b1 | 0.9792236 | 0.19923487 |
| 5033411D12Rik | 0.1757266 | 0.03575370 |
